# Acute myocardial infarction as the first presentation of thyrotoxicosis in a 31-year old woman - case report

**DOI:** 10.1186/1756-6614-3-1

**Published:** 2010-02-08

**Authors:** Krzysztof C Lewandowski, Tomasz Rechciński, Maria Krzemińska-Pakuła, Andrzej Lewiński

**Affiliations:** 1 Department of Endocrinology & Metabolic Diseases, The Medical University of Lodz, "Polish Mother" Memorial Research Institute, Rzgowska 281/89, 93-338 Lodz, Poland; 2 IInd Chair & Department of Cardiology, The Medical University of Lodz, The Bieganski Hospital, Kniaziewicza 1/5, 91-347 Lodz, Poland

## Abstract

A 31-year old woman, previously fit & well was admitted with pressing retrosternal chest pain and palpitations of sudden onset. Her body weight was normal (BMI 20.5 kg/m^2^) and there was no significant family history of cardiac disease. She smoked, however, about 15 cigarettes a day and she had been taking combined oral contraceptive pill (Cilest^®^) for about three years. On admission she appeared sweaty and in pain, blood pressure 130/70 mmHg, heart rate about 110/min, mild lid-lag sign. Heart sounds were normal and chest was clear. ECG revealed 2-3 mm ST segment elevations in II, III, aVF as well as V2 to V5. Troponin I was raised and she was qualified to an emergency coronary angiography. This revealed a massive spasm of left anterior descending (LAD) coronary artery that responded to intracoronary glyceryl trinitrite administration, however, with the presence of critical narrowing of the LAD apical segment with possible superimposed thrombus. Cardiac ultrasound revealed akinesis of 1/2 of apical area consistent with myocardial infarction

**Treatment and progress:**

She was started on Aspirin, Simvastatin, and Diltiazem, but continued to have persistent tachycardia and tremor. Thyroid function tests were ordered and showed thyrotoxicosis [free T4-46.9 pmol/l (ref. range 9-25), free T3-11.9 pmol/l (2-5), TSH - 0.02 mIU/l (0.27-4.2)]. She was referred for an endocrine opinion and started on Thiamazole. Other investigations revealed elevated anti-TPO and anti-TSH receptor antibodies consistent with Graves' disease. Thrombophilia screen was negative. She had remained euthyroid on a "block & replace" regimen (Thiamazole plus L-Thyroxine) that was discontinued after 18 months. She denies any anginal symptoms, but continues to smoke against medical advice.

**Conclusions:**

Our case highlights the possibility of development of an acute myocardial infarction in a young subject with thyrotoxicosis. We speculate that patient's smoking habit combined with subtle thyrotoxicosis-induced prothrombotic state and/or coronary-artery spasm had lead to the above-mentioned acute coronary event.

## Background

Acute myocardial infarction (MI) can occur in young subjects, and typical precipitating factors may include hyperlipidaemias, hypercoagulable states, cocaine abuse and other factors. According to the US data about 80% of young subjects are overweight or obese and at least one the classical cardiovascular risk factors is present in about 96% of cases [1]. Acute MI is, however, still relatively rare in young women and in a case series of 165 patients admitted with acute MI aged below 45 with (average age 41.3 ± 4.6 years, range 22-45 years) women constituted only 16% of cases [1]. We present the case where acute MI was an initial presentation of thyrotoxicosis in a young women of 31 years of age who had been entirely fit & well prior to this acute coronary event.

## Case Presentation

**Figure 1:**
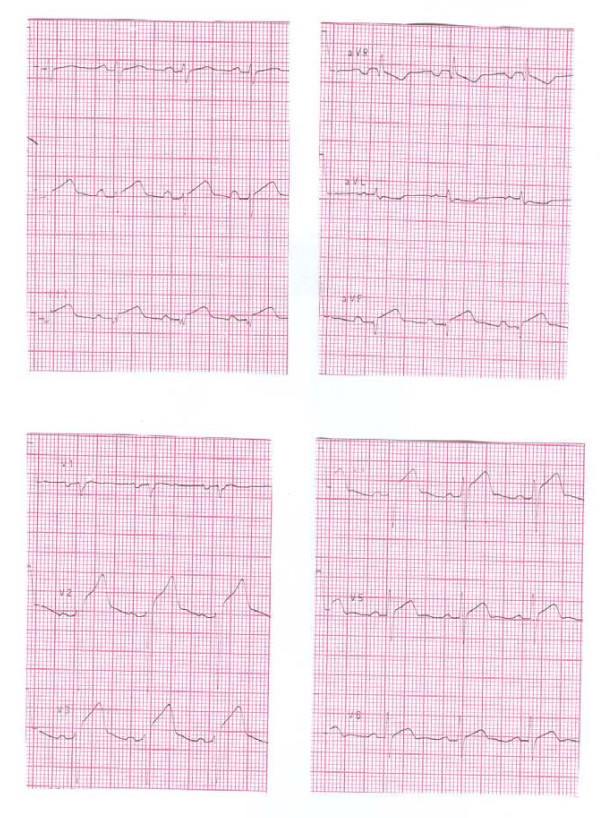
**Admission ECG of the presented patient with an acute myocardial infarction and thyrotoxicosis**. The tracing demonstrates 2-3 mm ST segment elevations in II, III, aVF as well as V2 to V5.

**Figure 2:**
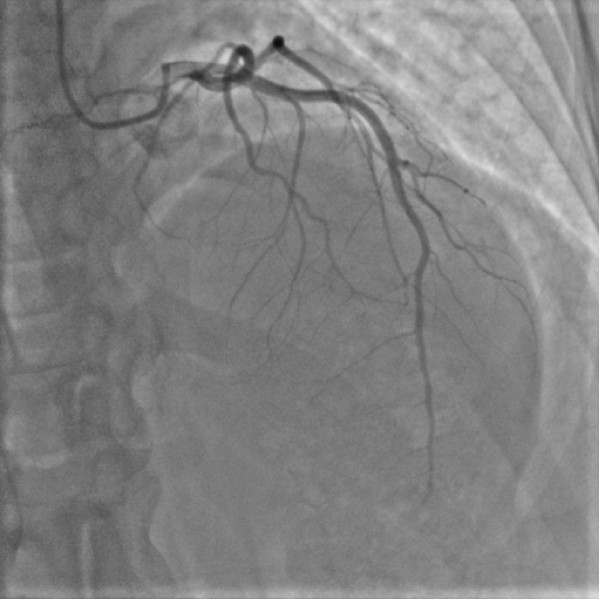
**Critical narrowing of left anterior descending artery in the presented patient close to the apical region with flow cessation possibly with residual thrombus, no evidence of significant narrowing in other vessels**.

**Figure 3:**
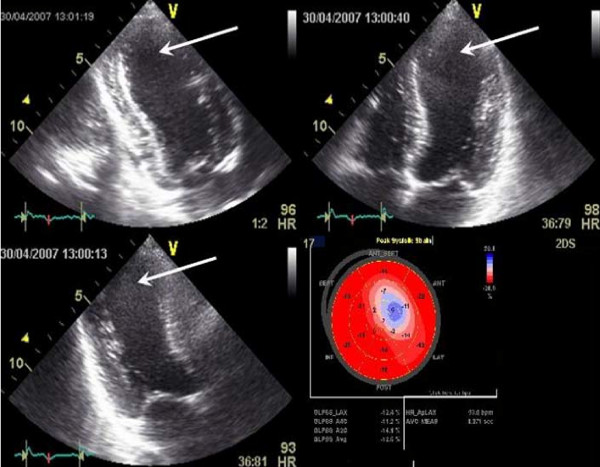
**Echocardiographic image showing left ventricular wall motion abnormalities of the apex in systole (arrows): apical two chamber view (left upper panel), four chamber view (right upper panel) and three chamber view (left lower panel)**. Matching bulls eye display of left ventricular segments strain quantitative analysis (right lower panel). Reduced myocardial contraction within the region of myocardial infarction is clearly visible (blue and pink colour).

A 31-year old woman, previously fit & well was admitted with a history of sudden onset of pressing retrosternal pain and palpitations. Her body weight was normal (BMI 20.5 kg/m^2^). She had no significant family history of ischaemic heart disease and had one healthy child of 5 years of age. She smoked, however, about 15 cigarettes a day and she had been taking Cilest^® ^i.e. a combined oral contraceptive pill, that contains 0.035 mg of Ethynylestradiol and 0.250 mg Norgestimate, for about three years prior to her admission. On examination she was sweaty and in pain, blood pressure 130/70 mmHg, heart rate about 110/min, mild lid-lag sign. Heart sounds were normal and chest was clear. ECG (see Figure 1) revealed 2-3 mm ST segment elevations in II, III, aVF as well as V2 to V5. Initial laboratory tests revealed normal electrolytes, and clotting screen, normal Full Blood Count and surprisingly low concentrations of plasma lipids (total cholesterol - 117 mg/dl, triglycerides - 93 mg/dl, LDL-cholesterol - 61 mg/dl, HDL-cholesterol - 37.8 mg/dl). Troponin I levels were, however, markedly raised (12.2 μg/l) and so she was qualified for an emergency coronary angiography (presented in Figure 2). This revealed a massive spasm of left anterior descending (LAD) coronary artery that responded to intracoronary glyceryl trinitrite administration, however, with the presence of critical narrowing of the LAD apical segment with possible superimposed thrombus. Cardiac ultrasound revealed akinesis of 1/2 of apical area with hypokinesis of adjacent segments, ejection fraction 38% - Figure 3. The titre of antinuclear antibodies was normal.

She was started on standard medication used in patients after myocardial infarction (Aspirin, Clopidogrel, Heparin, Simvastatin, Diltiazem), but despite this she was noted to have persistent tachycardia about 100-110 beats/minute and tremor. On the strength of that thyroid function tests were requested and revealed thyrotoxicosis (see Table 1). She was started on Thiamazole (Metizol©) 20 mg b.d.and referred for an endocrine opinion.

**Table 1:** Selected biochemical results and thyroid function test of the presented 31-year old female patient with an acute myocardial infarction

					Thyroid hormones		
Glucose (mg/dl) Ref: 60-100	Urea (mg/dl) Ref: 20-40	Creatinine (mg/dl) Ref: 0.5-1.35	Total cholesterol (mg/dl)	Triglycerides (mg/dl)	Free T4 (pmol/l) Ref: 9-25	Free T3 (pmol/l) Ref: 2-5	TSH (mIU/l) Ref: 0.27-4.2
94	21	0.42	117	93	46.9	11.9	0.02

In the Endocrine Clinic she was still hyperthyroid with a thyroid bruit and was clinically diagnosed to have Graves'disease. Thyroid ultrasound scan showed no focal lesions. She was subsequently found to have raised both anti-TSH receptor and anti-thyroid peroxidase antibodies that was confirmed autoimmune thyrotoxicosis (Table 2). Further autoimmune screen (also Table 2) revealed no evidence of thrombophilia. Following normalisation of free T4 and free T3 she was stabilized on a "block & replace regimen" (Thiamazole (Metizol©) plus L-thyroxine) that was continued for about 18 months. She currently remains euthyroid without medication [TSH - 1.39 mIU/l (Ref. 0.27-4.2)], but despite several warnings continues to smoke about 10 cigarettes a day. Following successful treatment of thyrotoxicosis her lipid profile showed significant rise in cholesterol levels in comparison to the thyrotoxic phase suggestive of occult dyslipidaemia previously masked by thyrotoxicosis (total cholesterol - 227 mg/dl, LDL-cholesterol - 164 mg/dl, HDL-cholesterol - 41 mg/dl, triglycerides - 109 mg/dl).

**Table 2:** Results of auto-immune screening of the presented 31-year old patient with a history of an acute myocardial infarction and thyrotoxicosis obtained in the Endocrine Clinic.

Thyroid peroxidase antibodies (aTPO) (IU/ml) [Ref.<60]	Anti-TSH receptor antibodies (IU/l) [Ref.<1.8]	Lupus anticoagulant (ratio) [Ref. ratio < 1.3]	Factor V (%) [Ref. 70-120]	Protein S (%) [Ref. 70-125]	Protein S (%) [Ref: 70-140]
607	22.8	1.05	77.1	71.3	104

## Discussion

Both hyper- and hypothyroidism are known to be associated with coagulation abnormalities. In case of hypothyrodism the data are somehow conflicting and the type of coagulation abnormalities may be, at least in part, related to the degree hypothyroidism [2]. In brief, milder forms of hypothyroidism may present with abnormalities resembling von Willenbrand's disease [3], i.e. decreased platelet adhesives, abnormal bleeding times, decreased levels of factors VIII, IX, XI and XII and a state with low von Willebrandt factor (vWF) activity [3], while severe hypothyroidism may result in a hypocoagulable state as shown by increases in platelet and factor VII as well as by decreases in fibrinolytic activity [4]. On the other hand there is quite consistent evidence that thyrotoxicosis is directly associated with the presence of a prothrombotic state. Horne et al. [5] demonstrated higher concentrations of prothrombin fragment 1 + 2, fibrynogen, factor VIII, antithrombin, tissue plasminogen activator antigen (tPA), plasminogen activator inhibitor 1 (PAI-1), PAI-1/tPA ratio and C-reactive protein in subjects with a history of cured thyroid cancer receiving standard TSH-suppressive L-thyroxine therapy in comparison to concentrations of the above parameters measured in the same subjects in a hypothyroid phase prior to radioiodine whole-body scanning procedure. Interestingly, anticoagulative factors, namely protein C and plasmin-antiplasmin complexes were also significantly lower during the hyperthyroid period. Squizzato et al. [6] described increased incidence of acute cerebral ischaemia in subjects with hyperthyroidism that was independent of thyrotoxic atrial fibrillation and cardioembolic stroke. More recently Homoncik et al. [7] have reported raised concentrations of vWF and increased baseline platelet plug formation in patients with thyrotoxicosis. These abnormalities were corrected by treatment of thyrotoxicosis with thiamazole. Also subjects with L-thyroxine-induced thyrotropin suppression as part of treatment of benign thyroid nodules were found to have a pro-coagulable profile [8]. In contrast, subjects with hypothyroidism had lower concentrations vWF antigen and factor VIII together with longer partial thromboplastin time (APTT) that was corrected by treatment with L-thyroxine. This might indicate the potential for increased bleeding tendency in subjects with hypothyroidism. We speculate whether the above mentioned abnormalities may contribute to an increased prevalence of menorrhagia typically observed in subjects with hypothyroidism.

Furthermore, long-term follow-up studies have revealed increased mortality from cardiovascular and cerebrovascular disease in those with past history of overt hyperthyroidism [9] as well as in those with subclinical hyperthyroidism [10]. In another study an elevated serum fT3 concentration was associated with a 2.6-fold greater likelihood of the presence of a coronary event [11].

Our subject was a smoker, and so despite a normal BMI she had a clear risk factor for CV disease i.e. like 96% of young subjects presenting with MI, as described, by Zarich et al. [1]. Though connective tissue disease [12] or factor V Leyden mutations and other thrombophilias have been linked with acute thrombotic events [13], we did not find evidence of thrombophilia or connective tissue disease in our subject. Her lipid profile with low cholesterol levels is likely to be a direct result of thyrotoxicosis, but one should note that satisfactory lipid profiles are not uncommon among young subjects with MI. For instance, Zarich et al. [1] reported LDL cholesterol levels below 100 mg/dl in 32% of young subjects with acute MI, while Akosah et al. [14] report LDL cholesterol levels below 130 mg/dl in more than 50% young adults with premature heart disease. Though our subject did use an oral contraceptive pill, we note that in contrast to increased risk of deep venous thrombosis, acute MI is actually very rare among the users of oral contraceptive pill and no evidence of thrombophilia [15]. In another recent study there was no increase in the risk of acute MI among 48321 Swedish women aged 30-49 years who used oral contraceptive pill [16]. Coronary artery spasm, also described in our patient, might be another factor contributing the the development of an acute myocardial infarction. There are also reports of severe coronary artery spasm leading to a myocardial infarction in young subjects with thyrotoxicosis, however, without classical cardiovascular disease risk factors [17]. Furthermore coronary artery spasm seems to be more common in smokers [18], while our patient smoked about 15 cigarettes a day.

Administration of iodine contrast during coronarography was also unlikely to have any adverse effects on thyroid function as iodine contrasts typically suppress thyroid hormone release in the short-term (*the Wolff-Chaikoff effect*), the phenomenon that is utilized in rapid preparation for thyroid surgery, or in the treatment of thyrotoxic storm [19,20]. Only prolonged administration of iodine compounds may contribute to the development of thyrotoxicosis through the so called Wolff-Chaikoff escape phenomenon [20].

## Conclusions

Our case highlights the possibility that thyrotoxicosis may facilitate development of an acute myocardial infarction even in a young person without previous history of thrombosis, vasculitis or heart disease. In our opinion cigarette smoking combined with a thyrotoxicosis-induced prothrombotic state was enough to tip the balance towards development of acute coronary thrombosis and subsequent acute myocardial infarction even in such a young and not-obese female subject. Our case therefore highlights the need for awareness of the possibility of such serious complication even among young subjects with thyrotoxicosis.

## Consent

Written informed consent was obtained from the patient for publication of this case report and any accompanying images. A copy of the written consent is available for review by the Editor-in-Chief of this journal.

## Competing interests

The authors declare that they have no competing interests.

## Authors' contributions

KCL (PhD MRCP) (UK): Principal management of the patient in the Endocrine Clinic, writing of the paper.

TR (PhD): Principal management of the patient in the Department of Cardiology, writing of the paper

MK-P (PhD Professor of Medicine & Cardiology): Senior supervision of the management of the patient in the Department of Cardiology (including cardiac ultrasound), writing of the paper.

AL (PhD Professor of Medicine & Endocrinology): Senior supervision of endocrine management, writing of the paper.

All authors have read and approved the final manuscript.
